# Acute Pancreatitis Complicating Acute Hepatitis E Virus Infection: A Case Report and Review

**DOI:** 10.1155/2013/531235

**Published:** 2013-01-31

**Authors:** Hemanta Kumar Nayak, Nitish L. Kamble, Nishant Raizada, Sandeep Garg, Mradul Kumar Daga

**Affiliations:** LN Hospital, Maulana Azad Medical College, New Medical Block, Room No. 121, New Delhi 110002, India

## Abstract

Acute pancreatitis complicating fulminant viral hepatitis has been well recognized; however, acute pancreatitis occurring in nonfulminant hepatitis is very rare. The case presented describes moderate pancreatitis in a young male, manifesting during the course of nonfulminant acute hepatitis E infection. The diagnosis of acute viral hepatitis E was confirmed by serology and reverse transcriptase polymerase chain reaction (RT-PCR) to demonstrate Hepatitis E virus (HEV) RNA in both stool and serum. Patients with acute viral hepatitis presenting with severe abdominal pain should have a diagnosis of acute pancreatitis suspected and appropriate investigations including serum amylase, lipase, biliary ultrasonography and/or contrast-enhanced computed tomography of the abdomen should be undertaken. The identification of this unusual complication of Hepatitis E is important; however, the prognosis for patients with Acute Pancreatitis Complicating Acute Hepatitis E Virus Infection is good, and uncomplicated recovery with conservative treatment is expected.

## 1. Introduction

*Background*. Patients with acute viral hepatitis who develop severe abdominal pain should be suspected of developing acute pancreatitis and appropriate investigations including serum amylase, lipase, biliary ultrasonography, and contrast-enhanced computed tomography of the abdomen to confirm the diagnosis indicated. The prognosis of patients with acute pancreatitis in the setting of acute viral hepatitis is good and patients recover with conservative treatment [1].

Acute pancreatitis is a common life-threatening disorder characterized by active inflammation of the exocrine pancreas associated with systemic inflammatory response. Clinical examination identifies the etiology of acute pancreatitis in more than 90% of cases with alcohol and cholelithiasis as the leading causes. Among other causes viral infections are well recognized. The viruses most frequently associated with acute pancreatitis are mumps, Coxsackie, rubella, Epstein-Barr virus, cytomegalovirus, and varicella-zoster virus. 

The association between infectious hepatitis and acute pancreatitis was first reported in 1944 by Linsey. Most cases are related to hepatitis A virus (HAV) and hepatitis B virus (HBV) infections. Furthermore there are a few reports of pancreatitis in acute hepatitis C and in exacerbations of chronic hepatitis B^.^ Hepatitis E virus (HEV) has recently been described as a causative agent of acute pancreatitis mostly in areas of endemic hepatitis E prevalence, but overt pancreatitis has only been reported generally in association with cases of fulminant hepatic failure. However recently it has come into focus that pancreatitis may also occur in hepatitis of mild-to-moderate severity.

## 2. Case Report

A 16-year-old boy was admitted with a history of jaundice of 8-days duration and recent onset of severe abdominal pain and vomiting. There was no history of alcohol use, gall stone disease, causative drugs, or trauma. In addition there was no family history or previous attacks of pancreatitis. He was an afebrile, but was jaundiced, and had tenderness over the epigastrium and umbilical regions and a palpable 1 cm liver edge. 

Laboratory reports revealed that abnormal liver function tests which include serum bilirubin 114.5 *μ*mol/L (normal 5.1–22 *μ*mol/L) (direct 63.2 *μ*mol/L, indirect 51.3 *μ*mol/L), AST 445 IU/L (Normal up to 40 IU/L), ALT 446 IU/L (Normal up to 40 IU/L), serum alkaline phosphatase 190 IU/L (Normal up to 170 IU/LIU/L), serum amylase 974 IU/L (normal range up to 170 IU/L), and serum lipase 645 IU/L (normal range up to 170 IU/L). Chest X-ray and ECG did not reveal any abnormality. Emergency ultrasound examination of abdomen revealed a bulky pancreas with hepatomegaly with no evidence of any cholelithiasis with nondilated common bile duct of diameter of 4 mm. Patient was treated conservatively.

On the fourth day of his hospitalization, repeat investigations revealed serum Bilirubin 201.7 *μ*mol/L (direct 179.5 *μ*mol/L, indirect 22.2 *μ*mol/L), AST 435 IU/L, ALT 565 IU/L, serum alkaline phosphate 290 IU/L, serum amylase 974 IU/L, serum calcium 2.25 mmol/L (Normal 2.2–2.6 mmol/L), phosphate 1.39 mmol/L (0.8–1.4 mmol/L), LDH 357 IU/L (Normal up to 300 IU/L), total serum proteins 74 gm/L (serum albumin 38 gm/L (Normal Albumin 40–60 gm/L), and globulin 36 gm/L (Normal 20–35 gm/L).

The increasing levels of jaundice and persistence of high level of liver-specific enzymes and serum amylase raised the possibility of viral hepatitis, and serum analysis identified raised IgM antibodies to hepatitis E virus. Reverse transcriptase polymerase chain reaction (RT-PCR) detected 10^8^ copies of HEV RNA/gm of stool samples and 10^5^ copies of HEV RNA/mL in blood samples. Serum lipid profile was found to be within normal limits. Contrast-enhanced computed tomography of abdomen done on the 5th day of illness revealed moderate pancreatitis and absence of peripancreatic collection. Gall bladder was of normal size with no cholelithiasis with normal common bile duct. 

## 3. Outcome and Followup

The patient was managed conservatively and recovered rapidly, and he was discharged after 9 days and remained asymptomatic when reviewed 4 weeks after discharge from hospital. Liver functions and serum amylase levels were within normal limits at this time. And rt PCR for HEV RNA was negative in both the stool and serum at 3-and 6-month followup.

## 4. Discussion

Viral causes of pancreatitis are well established. In adults mumps virus is one of the most commonly associated, etiological agents, and pancreatitis can occur even in the absence of parotitis [2]. In contrast, the association of viral hepatitis with pancreatitis is uncommon. Although a number of cases are described in association with fulminant hepatic failure [3]. It has been observed that pancreatic involvement may also be present in milder forms of viral hepatitis with a few a small number of reports describing mild-to-moderate pancreatitis associated with hepatitis E [1, 4–6].

The main case series are summarised in (Table 1), and in each series acute pancreatitis was seen more commonly in young males during the second and third weeks of the hepatitis illness and was usually self-limiting. Our case followed a similar course with moderate pancreatitis developing during the second week of Jaundice in a patient with hepatitis E proven serologically and by both stool and serum rt-PCR.

The incidence of pancreatitis in viral hepatitis is unknown. Postmortem studies have identified pancreatic inflammation in 12–40% of fatal hepatitis cases. 

 However, the association of pancreatitis with viral hepatitis is not a common clinical entity. A substantial number of these cases were found in association with fulminant hepatic failure [3]. It has recently been observed that pancreatic involvement may also be present in milder forms of viral hepatitis [1, 4]. There are only a few case reports from India of acute pancreatitis complicating mild-to-moderate hepatitis E virus infection [1, 5, 6]. 

The main case series are summarized in Table 1, and in each series acute pancreatitis was seen more commonly in young males during the second and third weeks of the hepatitis illness and was usually self-limiting. Our case followed a similar course with moderate pancreatitis developing during the second week of jaundice in a patient with hepatitis E proven serologically and by both stool and serum rt-PCR.

 The incidence of pancreatitis in viral hepatitis is unknown. Postmortem studies have identified pancreatic inflammation in 12–40% of fatal hepatitis cases [4]. Whilst work from the 1950's investigating the etiological factors of pancreatitis between 1 and 3.7% of patients with acute pancreatitis had a viral hepatitis [7]. 

The severity of pancreatic involvement in acute hepatitis may vary from mild to severe. Ham et al. in 1973 studied 42 autopsied cases of acute liver failure predominantly due to viral hepatitis cases and found all grades of pancreatitis [4]. The mechanism of pancreatic injury in viral hepatitis is unclear. Proposed mechanisms including oedema of the ampulla of Vater due to viral infection and associated acute liver failure have been suggested [8]. Rarely in sepsis by the effects of circulating bacterial products, mediated by cytokine release, also leads to pancreatitis. Recently, a direct cytopathic effect of the virus has been suggested, and the pancreas may also be a site of hepatitis B viral replication [9].

In most cases pancreatitis associated with benign forms of acute viral hepatitis resolve with little morbidity in conjunction with the recovery from hepatitis. However chronic relapsing forms of pancreatitis in association with viral hepatitis has been hypothesized [9]. Unusually severe abdominal pain early in the course of acute hepatitis should alert the clinician to the possibility of complications including acute pancreatitis and trigger appropriate investigations. However the prognosis of patients with acute pancreatitis in the setting of acute viral hepatitis is usually good and most patients recover with conservative treatment.

## Figures and Tables

**Table 1:** The summary of the data from three previous studies from India.

	Mishraet al. [1]	Jainet al. [5]	Bhagatet al. [6]
Total Number of patients	6	6	7
Age (mean)	13.5 yr	23.9	19.4
Gender (M : F)	5 : 1	7 : 0	6 : 1
HepA : HEPE	3 : 3	4 : 2	3 : 4
Method of confirmation of viral hepatitis	Serology	Serology	Serology
Day of onset of pancreatitis	10–22	2–30	3–17
Ser. bilirubin (mg%)	15.6	16.4	10.7
Ser. ALT (IU/L)	810	1371	484
Ser. amylase (IU/L)	795	365	1264
Ser. lipase (IU/L)	NA	2495	1382
Mortality	0	0	0
